# Study on the Application of Optical Current Sensor for Lightning Current Measurement of Transmission Line

**DOI:** 10.3390/s19235110

**Published:** 2019-11-22

**Authors:** Jin-ming Ge, Yan Shen, Wen-bin Yu, Yue Han, Fang-wei Duan

**Affiliations:** 1 Department of Electrical Engineering, Harbin Institute of Technology, Harbin 150001, China; gejinming90@126.com (J.-m.G.); ywb_hit@126.com (W.-b.Y.); 2 State Grid Liaoning Electric Power Research Institute, Shenyang 110006, China; hanyuehaha1@126.com (Y.H.); dfw8906@163.com (F.-w.D.)

**Keywords:** optical current sensing technology, lightning current measurement, transmission system

## Abstract

Accurate and reliable lightning current data are the basis of lightning protection design. To measure lightning current data at different measurement locations in a transmission system, the limitations of traditional lightning current sensors are analyzed, and optical current sensing technology is adopted, which has the advantages of no magnetic saturation and no bandwidth limitation. Compared with traditional application environments, the sensing technology is used in special environments in transmission systems. This paper analyzes the influence of environmental factors on sensors, and combines the extreme environmental requirements, such as temperature and insulation requirements, to study the sensor. Starting from the sensitivity, the sensing characteristics of the sensor are analyzed. The sensor is designed according to three aspects: sensing material selection, spatial measuring position, and sensing material size optimization, such that it can satisfy the different measurement requirements of towers, overhead ground wires, and transmission lines, respectively. The experiments indicate that the developed sensors can meet the measurement sensitivity requirements of different types of lightning strikes. The experimental results of sensors exhibit a reasonable amplitude measurement accuracy, linearity, and waveform measurement capability. These results provide important theoretical and experimental bases for the application of optical current sensing technology to the measurement of the lightning current of transmission systems.

## 1. Introduction

Lightning is a powerful natural discharge phenomenon that occurs between cloud-to-cloud or cloud-to-ground, of which the discharge process releases enormous energy and produces powerful electromagnetic pulses. Direct lightning strikes or lightning electromagnetic pulses not only pose a serious threat to human life safety, but also have a huge impact on the power grid, which, with wide-area distribution, leads to direct or indirect economic losses. The magnitude, steepness, and waveform of the lightning current play an important role in lightning protection design of the power grid [1]. As the distribution of lightning in different regions has a strong relationship with the local atmospheric conditions, topography, soil resistivity, and other factors [2], it is possible to investigate the lightning current parameter measurement statistics in different regions by installing lightning current measuring devices in a widely distributed transmission system. The lightning current parameter measurement statistics of areas are used to develop scientific differential lightning protection according to the lightning distribution and parameter characteristics of different regions [3]. Furthermore, lightning location systems (LLSs) are applied in lightning measurement and location in many areas; however, the direct measurement data of lightning current are necessary in order to calibrate the LLS using the lightning current sensor of a transmission system [4]. Therefore, it is very important to study lightning current sensors suitable for power transmission systems.

Some researchers have conducted extensive research in this field, and currently mainly use the following devices: magnetic steel bars [5], magnetic tape [6,7], and Rogowski coils [8,9]. Ping et al. [5] started using magnetic steel bars to measure the Zhejiang Xin-Hang line for several decades, on which the relevant industry standards in China are based. However, the measurement error of the magnetic steel bars is large; thus, it is not repeatable, and the installation and data reading method are inconvenient. The magnetic tape method significantly improves the measurement accuracy, stability, and ease of measurement compared with magnetic steel bars [6,7], but still cannot measure the lightning current waveform. The structure of the Rogowski coil is simple, convenient to install, and can be used to measure large pulse current signals with a large amplitude and rapid change. It has been a primary research target of lightning current measuring devices for transmission systems in recent years [8,9]. However, the Rogowski coil has a bandwidth limitation in the measurement principle, which contains low frequency information with large amplitude in lightning current. As a new type of current sensing technology, the optical current sensor (OCS) is only used in intelligent substations, because it has no iron core, no band limitation, and natural insulation, among others. It is also very suitable for lightning current measurement of transmission systems.

In this study, the application of optical current sensing technology, which can meet the waveform measurement requirements of lightning current, is taken as the research background. The different measurement requirements of the sensing system for the lightning current measurement of the transmission system are analyzed. Starting from the perspective of factors influencing the sensing characteristics of optical sensing materials, two types of OCSs suitable for power transmission systems were developed. Experimental platforms were also built to verify the sensing characteristics of the OCSs.

## 2. Design Idea of Lightning Current Sensor for the Transmission System

### 2.1. Sensing Model of OCS

**Figure 1:**
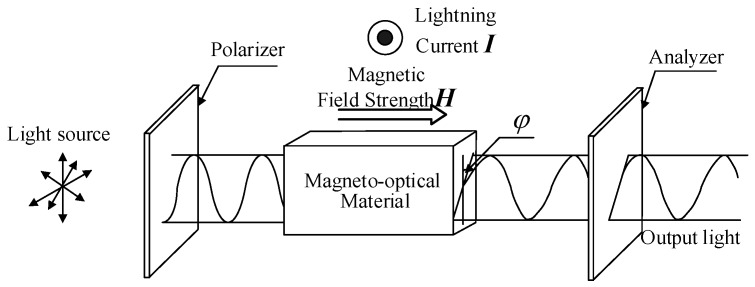
Faraday magneto-optical effect.

The Faraday magneto-optical effect sensing principle, applied by OCS for lightning current measurement, is shown in Figure 1. The Faraday rotation angle *φ* (rad) is expressed as

(1) dφ=VH⋅dl

where *V* is the Verdet constant (rad/T·m), *H* is the magnetic field strength (A/m), and *l* is the distance light passes through a magneto-optical material (m).

The relationship between *φ* and *H* in the light propagation direction can be expressed as

(2) $$\varphi =VHL=V\underset{L}{\int }H(L)dl$$

According to Malus’ law, the transmitted light intensity that passes through the analyzer can be described as follows:

(3) $$J_{o}=J_{i}\cos^{2}\alpha ,$$

where *J_i_* is the intensity of the polarized light (μw), α is the angle between the linearly polarized light and the analyzer (rad), and *Jo* is the output light intensity of the analyzer (μw).

Furthermore, the light intensity, which passes through the magneto-optical material and the analyzer, can be described as follows when linearly polarized light is rotated by a magnetic field:

(4) $$J_{o}=J_{i}\cos^{2}(\alpha -\varphi )=\frac{1}{2}J_{i}(1+\cos2(\alpha -\varphi )).$$

The sensitivity with which output light intensity can be used to estimate the Faraday rotation angle is as follows:

(5) $$\frac{dJ_{o}}{d\varphi }=J_{i}\sin2(\alpha -\varphi ).$$

Because *φ* is very small, let α=±45° to obtain larger responsivity to obtain the output light intensities *J_O_*_1_, *J_O_*_2_. The output of photoelectric conversion is obtained by dividing the difference between the electrical signals by the sum of them.

(6) $$u_{o}=\frac{J_{o1}-J_{o2}}{J_{o1}+J_{o2}}=\sin2\varphi$$

As seen in Equation (6), the influence of the light intensity fluctuation can be removed by processing the light path information, as described earlier.

According to the Biot–Savart law, for a straight wire of infinite length, the relationship between the magnetic field strength H at a certain point in the vicinity and the wire current *I* can be expressed as

(7) $$H=\frac{B}{\mu }=\frac{I}{2\pi r},$$

where *B* is the magnetic induction (T), *μ* is the permeability (H/m), and *r* is the distance between the point and the wire (m).

Substituting (7) into (2),

(8) $$\varphi =VHL=VI\underset{L}{\int }\frac{1}{2\pi r(l)}dl=KI,$$

where *K* is the sensitivity of the OCS, which contains all the factors that affect the rotation from the lightning current to the Faraday rotation angle. Therefore, by analyzing the sensitivity, we also analyze the sensing characteristics of the OCS. It can be seen from Equation (4) that *K* is related to the optical path length *L*; the magnetic field distribution, that is, the spatial position of the conduct and the OCS; and *V*, that is, the sensing material. Therefore, *K* can be expressed as

(9) K=f(a,V,L).

When the above three influencing factors are determined, the magnitude of the measured lightning current can be solved by the magnitude of the Faraday rotation angle *φ*. This paper will present the OCS suitable for lightning current measurement in transmission systems from the above three factors.

### 2.2. Environmental Requirements of Lightning Current Measurement

At present, the electronic transformers represented by OCS are mainly used in intelligent substations, and the installation locations are mainly located in GIS (gas insulated switchgear). The GIS equipment is completely filled with sulfur hexafluoride gas. All components in OCSs are in a fully enclosed state such that the influence of environment factors can be isolated, such as external water vapor and temperature. In the intelligent substation GIS, the current sensor is in an environment with good insulation performance; its overall space is small; and the distance between the sensor and the conductor is small, only about 0.2 m.

When the optical current sensing technology is applied in the lightning measurement of the transmission system, the measurement environment has no advantage in the intelligent substation. Therefore, it is necessary to study the following aspects: (1) Temperature. Because the lightning current measurement environment of the transmission system is in the wild, it is necessary to analyze the effect of temperature on magneto-optical materials. (2) Sensitivity. The wires in the transmission system are exposed in the air, and are suspended by the insulator on the tower. Therefore, when measuring the current on a three-phase transmission line, a certain safety distance needs to be met. In summary, in order to achieve the measurement of the waveform characteristics of the lightning current, when the OCS is applied to the environment of the transmission system, it is necessary to study and optimize the above aspects of the sensor for the actual situation of the extreme measurement environment.

### 2.3. Lightning Current Measurement Form in the Transmission System

In different types of lightning strikes, the main path that the lightning current flows through also differs in the transmission system. Therefore, lightning current measuring devices must be installed at different positions in the transmission system to measure the current of different types of lightning strikes. Table 1 presents a brief comparison of different measurement methods.

**Figure 2:**
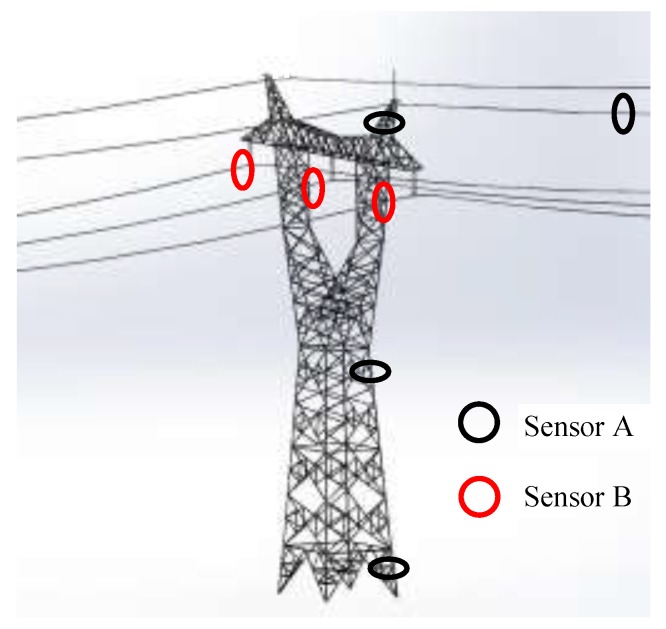
Sensor installation location diagram.

Table 1 indicates that, from the perspective of the lightning current sensor requirements on a transmission line, the sensor can be divided into two types: sensor type A represents lightning current measurement in a tower and in ground wires. All the measured currents of this type are at the ground potential and only need to be installed according to the use method of the measuring device at different positions where the lightning current passes. The insulation requirement for sensor A is not high; thus, it is installed close to the measured current using a magnetic steel bar or magnetic tape, and is directly installed on the conductors to be measured by a Rogowski coil. Sensor type B represents the lightning current measurement of phase conductors and must meet the insulation requirements. The use of such sensors should not damage the safe operation of phase conductors. Therefore, the sensor is required to realize noncontact lightning current measurement over a certain distance, such as a differential loop [15] or space measurement coil [17]. According to different measurement requirements, the installation position of the two types of sensors is shown in Figure 2.

**Figure 3:**
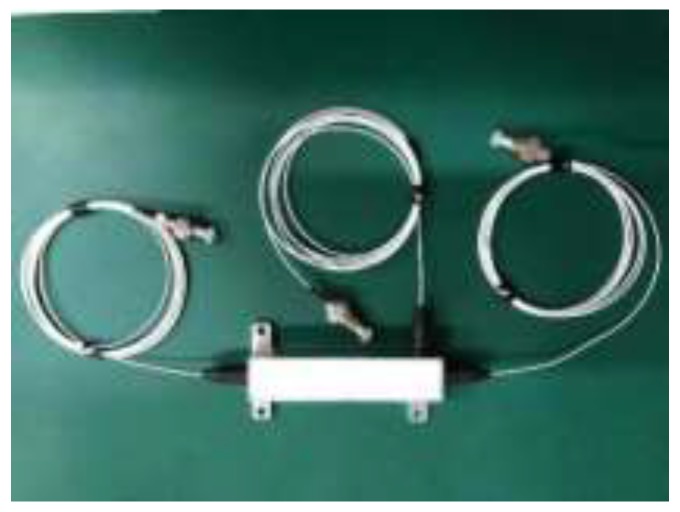
A photograph of the optical current sensing cell (OCSC) structure of the optical current sensor (OCS).

According to the above analysis, when the OCS is applied to the lightning current measurement of the transmission system, it is necessary to meet the lightning current measurement sensitivity requirements of different lightning strike types for the object to be measured. Compared with current transformers for substations, the lightning current measurement accuracy requirements are low and the measuring environment is complex, requiring high reliability of the sensor. In order to simplify the sensing structure as much as possible, the closed-loop feedback is discarded to ensure sufficient measurement accuracy, and the sensing performance is optimized from the design of the sensing unit. Therefore, the straight-through optical current sensing structure developed by our research group is used to research the sensing performance of the sensors in this study [16]. Figure 3 illustrates the structure of optical current sensing cells (OCSCs), which has a simple design, high reliability, uses dual-optical outputs to remove the influence of light intensity fluctuations, and is suitable for the harsh environment of lightning current measurement in transmission lines.

## 3. Magnetic Field Distribution of the Sensing Unit and Conductor Position

**Figure 4:**
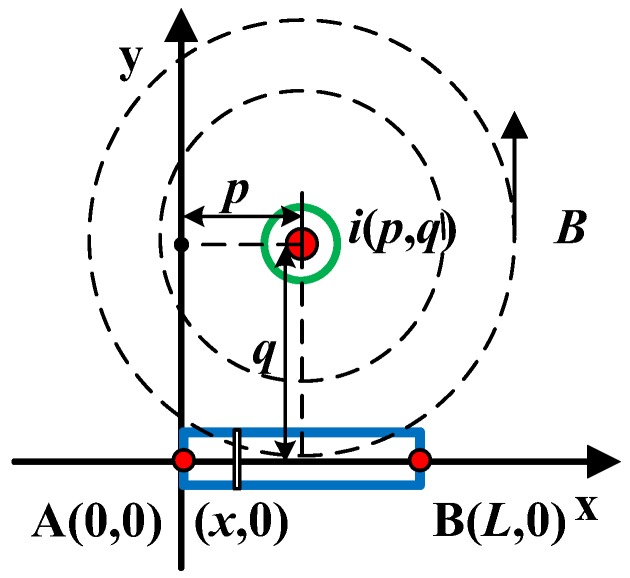
Relative position of the wire conductor to the sensor.

According to the basic principle of optical current sensing, the external magnetic field is a key factor causing the magneto-optical effect. If we set the optical path length to *L*, the relative position of the wire and the sensing unit is as shown in Figure 4, expressed as a=a(p,q).

In Figure 4, *p* is the distance of the wire from one end of the sensing unit and *q* is the vertical distance of the wire conductor from the sensing unit. The magnetic field component at any point on the sensing optical path that coincides with the direction of the optical path can be expressed as

(10) $$H(x)=\frac{I}{2\pi }\cdot \frac{q}{{\left(p-x\right)}^{2}+q^{2}}.$$

Corresponding to different effective magnetic fields, the obtained rotation angle can be expressed as

(11) $$\varphi =\frac{VI}{2\pi }\cdot \int_{0}^{L}\frac{q}{{\left(p-x\right)}^{2}+q^{2}}dx.$$

**Figure 5:**
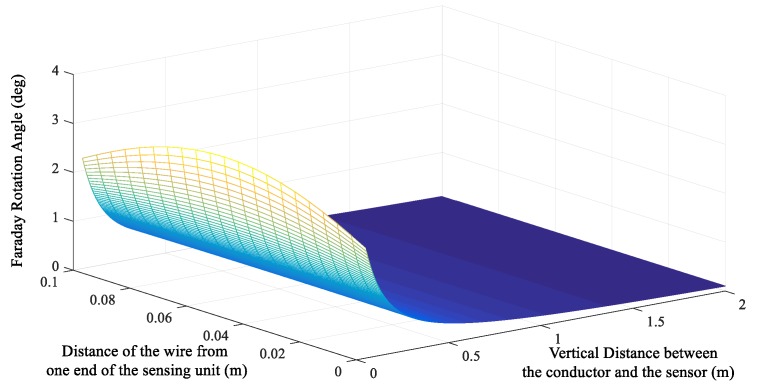
Faraday rotation angles in different relative positions.

It is assumed that the material is selected from dense flint glass model ZF-7, which has been widely used in the OCS in our group; its Verdet constant *V* = 17.8 rad/Tm, *I* = 10 kA, and *L* = 10 cm. When the temperature is constant and the linear birefringence caused by stress is not considered, the Faraday rotation angles in different relative positions are as shown in Figure 5.

It can be seen that, at a fixed distance, and as *p* increases, the rotation angle tends to decrease, and the Faraday rotation angle is at a maximum when the wire is at the center of the sensing unit. With the increase in *q*, the influence of the change in *p* on the rotation angle weakens. After exceeding 0.4 m, it does not vary with *p*, and the response of the sensor gradually decreases.

Sensor A has no insulation requirements and can achieve a large response by using a small installation distance. Therefore, the conductor should be installed at the center of the sensor, where a=a(L2,q), to get the maximum magnetic field. Sensor B must maintain a certain safe distance from the conductor to be measured. On the basis of the satisfaction of *q*, the value range of *p* is large, which also provides a large elastic margin for the long-distance installation accuracy of the sensor, which is applicable to the actual measurement of lightning current in the transmission system.

## 4. Research on the Sensing Unit of OCS

### 4.1. Determination of Sensing Material

It can be seen from the above analysis that the sensitivity of the sensor required by different measurement methods also differs. Equation (8) indicates that the sensitivity of the sensing unit can be satisfied by selecting different sensing materials. The magneto-optical materials can be divided into two types: diamagnetic and paramagnetic. The different responses when different materials are in the same magnetic field are described by the Verdet constant, which can reflect the sensitivity of the magneto-optical effect rotation angle to the external magnetic field, the value of which is related to the electronic layer structure of atoms or ions in a magneto-optical medium.

The ions in the diamagnetic material exhibit an inert gas electron layer structure when subjected to an applied magnetic field, and thus have no permanent electron orbital magnetic moment. Under the action of the magnetic field, only Zeeman splitting of the ion level can occur, causing a change in the electron orbital motion. The atom produces a small magnetic moment in the opposite direction, rotating the plane of polarization of the light wave passing through the medium in the magnetic field [18]; thus, its Verdet constant value is relatively small. Borrelli et al. derived the expression of the Verdet constant of the diamagnetic material based on quantum theory [19]:

(12) $$V=(4\pi Nv^{2})\sum \left[A_{n}/{\left(v^{2}-v_{n}^{2}\right)}^{2}\right],$$

where *N* is number of carriers per unit volume (mol/L); *A*_n_ is a parameter related to the strength of the transition; and *v* and *v*_n_ are frequencies of the incident light and electronic transition, respectively (Hz).

In paramagnetic materials, because of the presence of ions that are prone to transition, such as iron ions in the OCSahedral position in a Y_3_Fe_5_O_12_ (YIG) magneto-optical crystal, the rare earth ions Pr^3+^, Ce^3+^, Tb^3+^, Dy^3+^, and so on, contained in the paramagnetic glass electrons in the medium, will migrate when an external magnetic field is applied to the medium, indicating that linearly polarized light will produce a larger optical rotation angle in the medium. The Verdet constant of paramagnetic materials is larger than that of diamagnetic materials, and the sensitivity of the magneto-optical effect is higher. On the basis of quantum theory, the expression of the paramagnetic Verdet constant is [20]

(13) $$V=(K/T)(Nn_{eff}^{2}/g)\underset{n}{\sum }\left[C_{n}/{\left(v^{2}-v_{n}^{2}\right)}^{2}\right],$$

where $K=4\pi^{2}\mu_{B}v^{2}/3chk$, $n_{eff}=g{\left[J(J+1)\right]}^{1/2}$, *N* is the number of paramagnetic ions per unit volume (mol/L), *g* is the Lande splitting coefficient, *J* is the total angular momentum quantum number (mol), *C*_n_ is the transition probability, $\mu_{B}$ is the magnetic moment (A/m^2^), *c* is the speed of light (m/s), *h* is Planck’s constant (J·s), *k* is Boltzmann’s constant (J/K), and *T* is the absolute temperature (K).

For OCS type A, because there is no installation limitation, a large sensitivity can be obtained by a small installation distance. Therefore, diamagnetic magneto-optical glass with better stability is preferred as the sensing material. Model ZF-7 magneto-optical glass is used in this study as a sensing material for sensor A, for which the typical Verdet constant is 17.8 rad/Tm.

In the lightning current measurement of transmission lines, the OCS prioritizes the use of a larger-sensitivity paramagnetic magneto-optical material. In this study, a Tb_3_Ga_5_O_12_ (TGG) crystal is used as the sensing material. The crystal structure can achieve cation replacement in a larger ionic radius. Compared with representative paramagnetic materials such as Tb^3+^-doped magneto-optical glass and a YIG crystal, the crystal has a higher Verdet constant, as the typical Verdet constant is 134 rad/Tm in 633 nm; has a low absorption coefficient; and is highly transparent in the visible and near-infrared spectral range [21,22].

### 4.2. Temperature Compensation Method for OCS Type B

Equations (12) and (13) indicate that the Verdet constant of the diamagnetic material is a temperature-independent quantity, while the paramagnetic material is temperature-dependent. The lightning current measurement of the transmission system is operated outdoors, of which the ambient temperature will have a certain impact on the response of sensor B. In the case of high requirements for measurement accuracy, it is necessary to compensate for the material response fluctuation caused by temperature. The relationship between the Verdet constant and temperature can be obtained through the Verdet constant model of paramagnetic materials, and the corresponding temperature compensation value can be obtained according to the measured temperature.

When the wavelength of incident light is constant, the Verdet constant of the paramagnetic material as a function of ambient temperature *T* can be expressed as [23]

(14) $$V(T)=A\cdot (1+\frac{B}{T-T_{c}}),$$

where *T_c_* is the Curie temperature of the material and *A* and *B* are constants related to the properties of the material.

The response output signal of the OCS can be expressed as φ=K(T)I. The sensor was calibrated to temperature *T*_0_, and the ratio error caused by the change in temperature is

(15) $$\varepsilon =\frac{K(T)I-K(T_{0})I_{0}}{K(T_{0})I_{0}}.$$

When *T* = *T*_0_ is considered, and *ε* = 0, the temperature-induced ratio error can be obtained as

(16) $$\Delta \varepsilon =\left[\frac{K(T)}{K(T_{0})}-1\right].$$

The temperature fluctuation problem caused by the Verdet constant is equivalent to superimposing a temperature-dependent interference signal on the Faraday rotation angle Δφ(T). The temperature compensation method superimposes a compensation signal with the same value and negative relation with a sign on the sensing signal in the signal processing unit. This process can be expressed as

(17) $$\varphi (T)=\varphi_{0}+\Delta \varphi (T)+\varphi_{c}.$$

The error in response to temperature fluctuation is

(18) $$\Delta K(T)=K_{0}\Delta \varepsilon .$$

The temperature compensation signal can be obtained as

(19) $$\varphi_{c}=-\Delta \varphi (T)=-K_{0}I\Delta \varepsilon .$$

## 5. Research on Sensing Unit Size of OCS

It can be seen from Equation (8) that, the longer the length of the sensing material, the larger the Faraday rotation angle. It should be noted that the above formula was obtained under an ideal environment. Linear birefringence occurs with changes in fields such as stress and temperature, so linear birefringence must be considered when analyzing the size of magneto-optical materials.

(20) φ=η(L)[α(L)+k(L)·KI]

(21) $$\eta (L)=e^{-L\cdot \alpha_{0}}\cdot [1-0.85\frac{L\cdot \tan\beta }{f\cdot (NA)}]\cdot [1-0.5\frac{L\cdot a}{f^{2}\cdot (NA)}]$$

Equation (21) is the optical coupling efficiency of the optical current sensing unit, where $\alpha_{0}$ is the percentage of luminous flux absorbed by the white light passing through every centimeter of the magneto-optical material and initial luminous flux, *a* is the fiber radius, β is the tilt angle of the fiber axis, *f* is the lens focal length, and *NA* is the numerical aperture of the optical fiber.

(22) $$\alpha (L)=\frac{1}{2}\sin^{2}(\frac{\delta_{0}L}{2})\sin4\theta$$

Equation (22) is the zero drift angle, which is independent of the Faraday rotation angle. When there is linear birefringence in the OCS, even if the current is zero, the Faraday angle will also exist, where *δ*_0_ is the birefringence angle per unit length and *θ* is the incident azimuth of polarized light. By appropriately setting the incident azimuth of polarized light, the influence of the zero drift on the Faraday rotation angle can be eliminated.

(23) $$k(L)=\frac{\sin(\delta_{0}L)}{\left(\delta_{0}L\right)}$$

Equation (23) is the birefringence factor, which is the result of the presence of birefringence directly acting on the Faraday angle. The larger the value, the higher the sensitivity of the sensor. 

**Figure 6:**
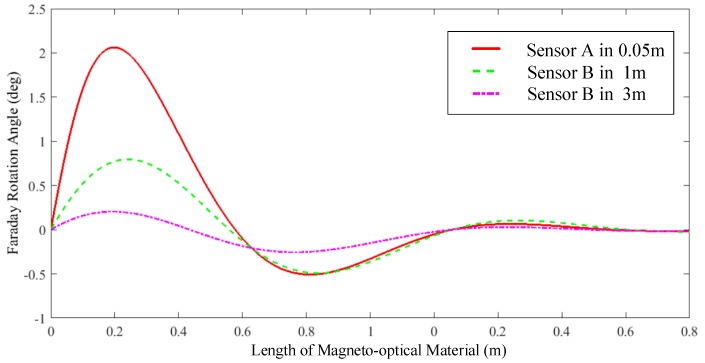
Relationship between the Faraday rotation angle and sensing material size.

Simulate the response of sensors with different lengths. Let tilt angle of the fiber axis *β* = 0.3°. The two types of OCSs are placed at positions 0.05 and 1 m away from the conductor, respectively, through which 10 kA of current flows. The simulation curve of the relationship between the Faraday rotation angle and the sensing unit size is shown in Figure 6.

It can be seen that, considering the optical coupling efficiency and linear birefringence, the Faraday rotation angle increases with the length of the sensing material and reaches a maximum after saturation. After that, the rotation angle decreases with the length. Considering the error caused by linear birefringence and the utilization of magneto-optical materials, the material length is selected from the optimal length, that is, the portion where the rotation angle increases fastest with the length before reaching its saturation. As the measuring distance increases, the optimal length also changes, but after the distance increases to a certain extent, the response change has a weak effect. This study selects two types of magneto-optical materials with a length of 5 cm.

It should be noted that the rotation angle of sensor B, which uses the magneto-optical crystal, is close to the amplitude of sensor A. However, because of the long distance from the conductor to be measured, the length of the magneto-optical material required for the rotation to reach saturation increases. In order to emphasize this influencing factor, we set the measuring point 3 m from the conductor. It can be seen from the rotation angle curve that, when the measuring distance is further increased, the curve becomes flat. The effect of material length on the degree of saturation of the rotation angle becomes weak. In addition, the effect of the length of the material caused by the change in distance on the saturation of the rotation angle also becomes weak, and the range of optimal lengths that can be selected is the same. Therefore, the principle of the length selection of magneto-optical materials has universal applicability under different measurement distance requirements.

## 6. Experimental Verification

### 6.1. Sensitivity Experiment of Magneto-Optical Materials

**Figure 7:**
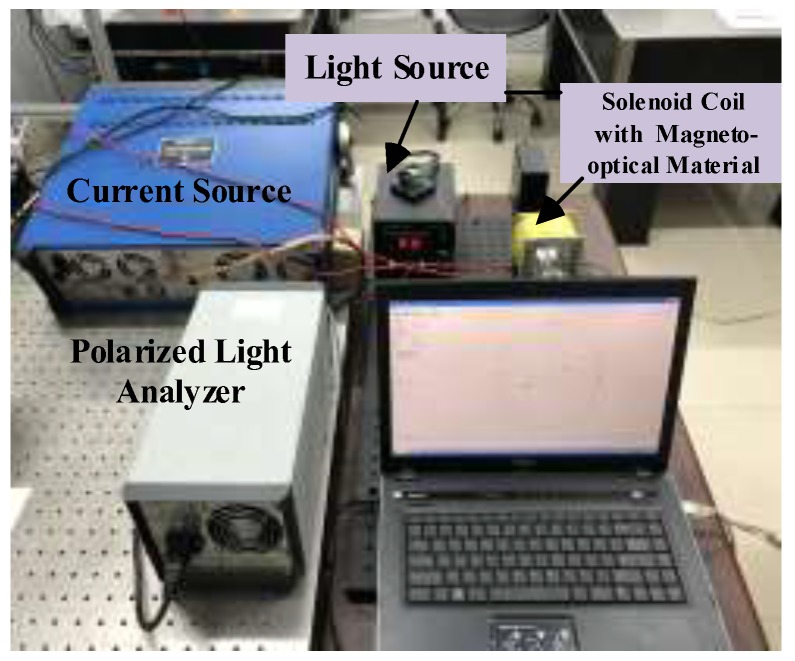
Schematic of experimental setup.

**Figure 8:**
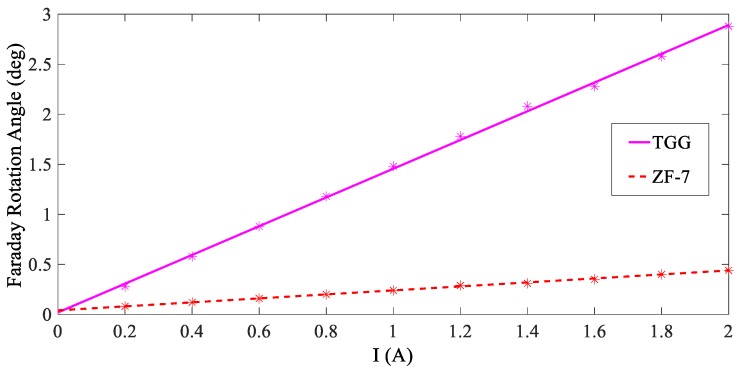
Response of two materials under different magnetic fields. TGG, Tb_3_Ga_5_O_12_.

In order to verify the sensitivity characteristics of the two types of magneto-optical materials, ZF-7 and TGG, a Faraday magneto-optical effect platform is used, as shown in Figure 7. After the light source passes through the collimator and polarizer, it becomes polarized. The polarized light is transmitted through the magneto-optical material and is deflected by the magnetic field, which is generated by the current flowing through the solenoid, which has a winding number of 630 and a resistance of 3.85 Ω. The uniformity of the magnetic field at the center of the solenoid is good. The modulated polarized light is detected by the polarizing light analyzer by which the rotation angle can be directly detected. We apply a direct current of 0–2 A to the solenoid and obtain ten voltage response values. The experimental results are presented in Figure 8.

From the figure, the response of both materials increases with the magnetic field, and both demonstrate a good linearity. The increase in the response reflects the sensitivity of the magneto-optical material. By function fitting, the relationship between the response of the two different materials and the applied current in the experimental system is obtained:

(24) $$\left\{ \begin{array}{l} \varphi_{\mathrm{ZF}-7}=0.199I+0.04\\ \varphi_{\mathrm{TGG}}=1.436I+0.02 \end{array}\right.$$

It can be seen that the slope of the two sets of curves can reflect the sensitivity of the system output to the excitation current when using different sensing materials. When using TGG, the slope of the function is about seven times that of ZF-7. The experimental results indicate that both materials can reflect the magnitude of the magnetic field through the rotation angle. The sensitivity is higher when using the TGG crystal, which is advantageous when applied to the lightning current measurement of transmission lines with the measurement distance requirements.

### 6.2. Temperature Characteristics Experiment of Magneto-Optical Materials

**Figure 9:**
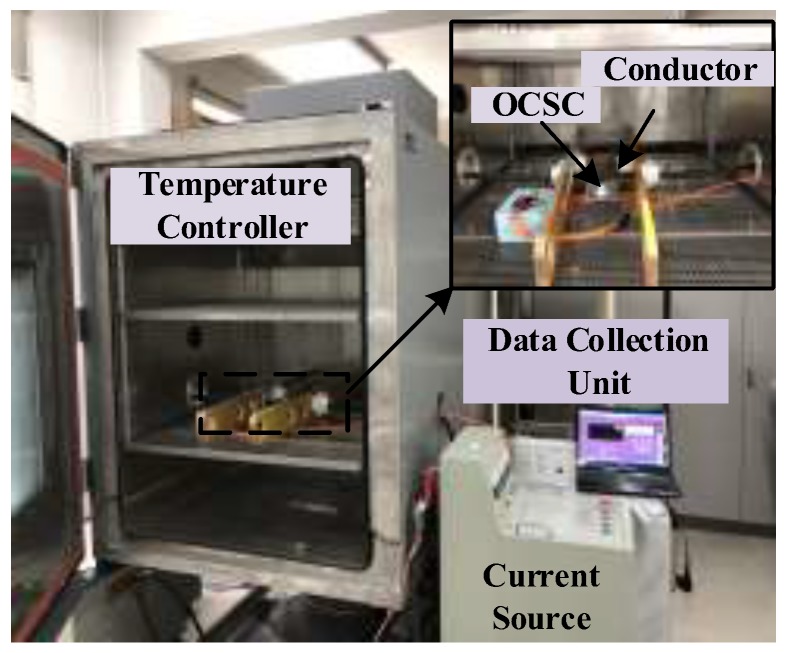
Schematic of temperature experimental setup.

**Figure 10:**
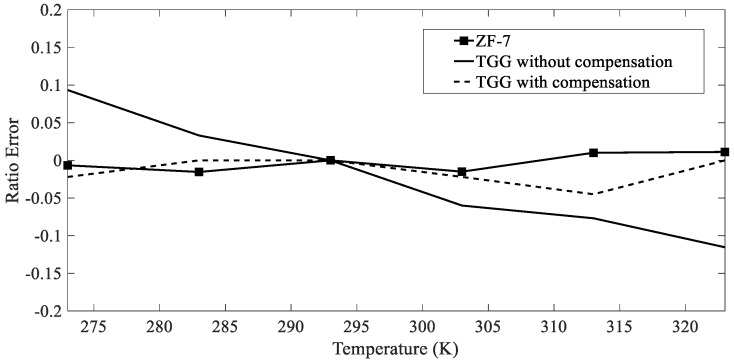
Effect of temperature on measurement error of magneto-optical materials.

The temperature experiment platform is set up as shown in Figure 9. The magneto-optical material is placed in a temperature controller, and the sensor is calibrated to room temperature of 298 K. We set the experimental temperature range to 273–323 K, and with every 10 K increase in temperature, it is kept warm for 1 h to ensure a uniform temperature effect on the material, and a magnetic field is generated by a power frequency current with an amplitude of 600 A. The effect of the temperature measured at each temperature point on the ratio error is shown in Figure 10. 

From the figure, the absolute value of the ratio difference of the ZF-7 magneto-optical glass in the temperature range is maintained within 0.04. The measurement result is only affected by the temperature-induced linear birefringence, where the fluctuation is sufficiently small to meet the measurement requirement. The Verdet constant and linear birefringence of the TGG magneto-optical crystal are affected by temperature, the ratio difference is approximately linear with temperature, and the fluctuation is relatively large. The temperature in thunderstorms during different seasons varies from region to region, but all are seasonally distributed [24,25]. The temperature change in the same region is relatively small in the short term, and the ratio error will decrease further after calibration. The error is much smaller than the average errors of the LLS (20%), which can achieve calibration of the LLS. If there is a further requirement for measurement accuracy, the temperature compensation method proposed in this paper can be used to compensate the temperature error of the Verdet constant of the TGG crystal. The ratio curve of each temperature point after compensation is similar to the fluctuation range of the ZF-7 ratio curve, where the temperature stability significantly improves.

### 6.3. Impulse Current Measurement Experiment

**Figure 11:**
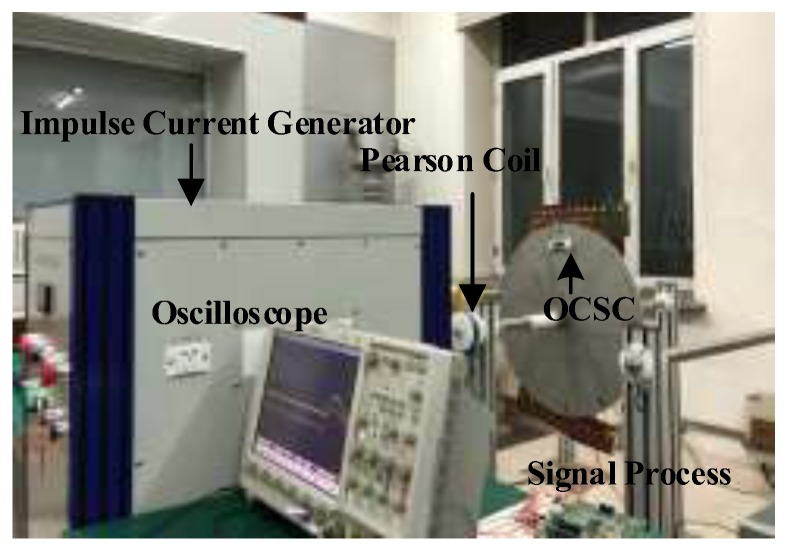
Impulse current measurement experimental platform.

**Figure 12:**
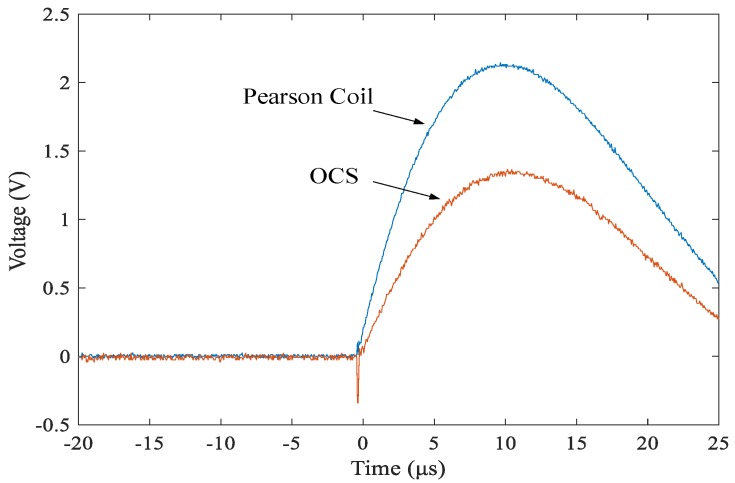
Current waveform measured by OCS.

We set up the impulse current measurement experimental platform (Figure 11). The lightning current source generates an impulse current with a waveform of 8/20 μs. The measurement results were calibrated using a Pearson current sensing head. The measurement locations of the two sensor types were set to 0.05 and 0.5 m, respectively. The measurement result of sensor A is taken as an example to illustrate the impulse current waveforms of the OCS, which is shown in Figure 12.

From the figure, the output waveform is consistent with the lightning current source waveform, indicating that the sensor proposed in this paper can accurately reflect key information such as the amplitude, steepness, and waveform of the lightning current flowing through the conductor. It should be noted that the shape and current path of the conductor may affect the measurement results; it can be calibrated according to different conductor conditions in the actual application. The lightning current of different amplitudes was measured multiple times at the same position. The response of the two sensor types is presented in Table 2.

As can be seen from the above figure, the measurement waveform of OCS is consistent with that of the person coil, which can meet the requirements of type A for accurate full waveform measurement, and can also meet those of type B measurement of key information such as amplitude and steepness. The amplitude of the impulse current is changed under the same experimental condition, and the responses of the two types of sensors are as shown in Table 2.

Table 2 indicates that, with the increase in the peak value of the impulse current, the outputs of sensors have linearity and stability. The mean sensitivity values are 0.637 and 0.475, respectively, although the measuring distances are quite different between the two sensor types. The response of the sensor is comparable and consistent with the theoretical analysis. Referring to the existing waveform peak test results, using the optical current sensing principle, that is, in the current measurement of hundreds kA amplitudes, also has a good measurement effect [26]. Combined with the better linearity of the impulse current measurement, it can be illustrated that optical current sensing technology is applicable in high amplitude peak current measurement.

## 7. Conclusions

Accurate lightning current parameters form the basis of lightning protection designs of power systems. On the basis of the analysis of characteristics of traditional lightning current measuring devices in the transmission system, this study applies the optical current sensing technology to lightning current measurement. Two types of lightning OCSs were studied, and the results were verified by experiments.

1. On the basis of the analysis of the measurement of the existing lightning current measuring device of the transmission system, the sensing characteristics of the OCS were analyzed from the perspective of sensitivity. The ZF-7 and TGG crystals were used as the sensing materials to meet the transmission measurement requirements of different types of lightning strikes.
2. It is verified by experiments that the sensitivity characteristics of the two magneto-optical materials are suitable for the lightning current measurement in the transmission system. With the temperature compensation method proposed in this paper, the two types of OCSs can maintain a reasonable measurement accuracy under the lightning temperature environment of the transmission system.
3. When the two types of sensors are at different measuring distances, and the impulse currents of different amplitudes flow through the conductor to be measured, the measured amplitudes of the two types of OCSs can maintain a good linearity, which illustrates the applicability to different measurement distance requirements.

In this study, the sensing characteristics of different magneto-optical materials were utilized, which provides a reference for the application of new sensing materials. In addition, the results presented in this paper provide research ideas for the design of lightning current waveform measuring devices that meet different measurement requirements such as wind power and aviation. Subsequent research can be carried out in the practical direction of two types of OCSs:

1. For sensor A, the distortion of the measured lightning current is small and can be used to estimate the original parameters of the lightning current; however, the measuring current is affected by factors such as the material and height of the tower. The distortion factors can be researched to obtain the true lightning current parameters.
2. For sensor B, the measurement target is the lightning current transmitted from the strike position; thus, the characteristics of the measuring current can be studied to realize the functions of lightning strike point location and lightning strike fault recognition.

## Figures and Tables

**Table 1:** Comparison of different measurement methods.

Measured Lightning Strike Type	Type of Fault Caused	Sensor Installation Position
Lightning strike at the top of a transmission tower	Flashover caused by back striking	Tower top, tower body, tower foot, centers of the intervals on the ground wires, and ground wire bracket [10,11,12,13,14]
Lightning strike on a transmission line	Flashover caused by shield failure	
Lightning strike on overhead ground wires	Shielding failure or flashover caused by back striking	Transmission line [15,16]
Inductive lightning	Invading wave of substation	

**Table 2:** Response of the optical current sensor (OCS).

Measuring Distance (m)	I (kA)	U (V)	S (V·kA^−1^)
0.05 (Sensor A)	1.75	1.11	0.634
	2.13	1.358	0.638
	4.26	2.726	0.64
	5.37	3.43	0.639
0.5 (Sensor B)	1.73	0.817	0.472
	2.52	1.207	0.479
	3.85	1.828	0.475
	4.96	2.35	0.473
